# Communication Experiences and Challenges in Adult Cochlear Implant Users: Associations with Age and Occupation

**DOI:** 10.3390/jcm15041450

**Published:** 2026-02-12

**Authors:** Jack Y. Lin, Hugh M. Birky, Aaron C. Moberly, Terrin N. Tamati

**Affiliations:** 1 Vanderbilt University School of Medicine, Nashville, TN 37232, USA; jack.y.lin@vanderbilt.edu; 2 Department of Otolaryngology—Head and Neck Surgery, Vanderbilt University Medical Center, Nashville, TN 37232, USA; hugh.m.birky@vumc.org (H.M.B.);; 3 Department of Speech and Hearing Science, The Ohio State University, Columbus, OH 43210, USA

**Keywords:** cochlear implants, cochlear implant outcomes, speech perception, communication, age, occupation, rehabilitation

## Abstract

**Background/Objectives**: Over one million individuals have received cochlear implants (CIs) worldwide—a monumental milestone in improving speech perception for those otherwise unable to hear. Although implantation is now routine, communication outcomes vary widely. This study investigated the effects of age and occupational status on the communication challenges of postlingually deafened adult CI users. **Methods**: Sixty-nine experienced (>6 months of use) CI users between the ages of 18 and 83 years completed a lab-developed survey. Self-reported communication challenges were compared between younger (<65 years) and older (≥65 years) CI users, and between working and retired individuals. **Results**: Younger CI users placed greater importance on communicating with colleagues than older CI users (*p* = 0.001), a pattern also observed among those working compared with retired individuals (*p* < 0.001). Compared with older CI users, younger participants reported fewer difficulties understanding fast (*p* = 0.012) and unclear speech (*p* = 0.016), but greater difficulties with soft (*p* = 0.044) and foreign-accented speech (*p* = 0.047). Similarly, working CI users reported fewer difficulties with fast (*p* = 0.028) and unclear speech (*p* < 0.001). Regardless of age or occupational status, most participants reported persistent listening fatigue and the tendency to avoid difficult conversations. **Conclusions**: Together, these findings demonstrate that while adult CI users report common struggles like fatigue, specific communication challenges differ across age and occupational status. Recognizing these factors may inform more personalized counseling and rehabilitation strategies to enhance everyday communication outcomes for CI users.

## 1. Introduction

Cochlear implants (CIs) have become an increasingly common intervention for moderate-to-profound hearing loss, with more than one million individuals implanted worldwide since the first procedure in 1961 [1,2]. This number represents a considerable jump from 60,000 CIs in 2004 to 736,900 in 2019 [3,4]. The World Health Organization (WHO) further estimates that 2.5 billion people will have some degree of hearing loss by 2050, with over 700 million requiring hearing rehabilitation [5]. Given the current and projected growth in CI usage, it is critical to consider how to optimize functional hearing and communication outcomes.

Adult CI users show wide variability in outcomes, which are typically assessed using clinical speech recognition measures such as AzBio sentences and CNC words [6,7,8]. Among CI users, speech recognition scores have been shown to vary according to demographic, audiological, surgical, and device-related factors [8,9,10]. Patient-specific experiential and environmental factors—such as musicality, crystallized and fluid intelligence, and socioeconomic status—may also contribute to variability in CI outcomes [11,12,13]. However, conventional clinical speech recognition outcome measures do not fully capture the lived experiences of CI users [14,15,16,17]. Many adult CI users continue to report a wide range of real-world difficulties, such as understanding speech-in-noise, understanding certain communication partners (e.g., those with fast or accented speech), listening fatigue, and social disconnection [18,19,20,21,22,23,24,25,26]. The specific everyday challenges experienced by adult CI users are also likely to be shaped by a combination of individual and environmental factors. However, relatively little is known about how age and occupational status might influence subjective communication experiences.

Prior research has shown that advancing age may play a role in speech perception outcomes and quality of life in CI users [27,28,29]. For example, Bourn et al. (2022) [28] found significantly poorer AzBio sentence recognition scores among adults aged ≥80 years compared to younger adults aged 65–79. While older adults often demonstrate poorer behavioral speech recognition performance [30,31,32], how age impacts subjective communication experiences and challenges remains less clear. Broader patterns of social behavior may contribute to these impacts: younger adults often maintain more extensive and dynamic social networks, engage in higher-frequency interactions, and tolerate greater cognitive load, whereas older adults may encounter more social isolation and limited communication opportunities [33,34,35,36]. These differences suggest that age may shape both the types of challenges CI users experience and the priorities they place on overcoming them.

Age-specific differences in communication behaviors may be driven, at least in part, by associated changes in occupational status. For example, McRackan et al. (2019) [27] found that being employed was positively associated with communication, listening effort, and social CI quality of life domains. However, these findings were based on composite scores across multiple questions within broad domains, rather than specific communication contexts. Employment may expose CI users to unique challenges—such as frequent group discussions, meetings, or noisy environments—that differ substantially from those faced by retired individuals, who may instead contend with smaller social networks and limited patterns of interaction [37,38,39,40]. Thus, occupational status may shape the subjective communication experiences and challenges of adult CI users.

Given these gaps in knowledge, the objectives of this study were twofold: (1) to define the self-reported communication experiences and challenges of experienced adult CI users and (2) to compare communication experiences and challenges between age—younger (<65 years) vs. older (≥65 years)—and occupational status (working vs. retired) groups. We hypothesized that adult CI users would report a broad spectrum of self-reported communication experiences and challenges, with both shared and distinct patterns emerging according to age and occupational status.

## 2. Materials and Methods

### 2.1. Participants

A total of 76 adult, postlingually deafened CI users participated in the current study. After the exclusion of 7 incomplete survey responses, a sample of 69 CI users who were between the ages of 18 and 83 years (Mean = 61.6, SD = 13.3) were included in the analysis. Data were collected as part of two remote-testing studies on CI speech communication outcomes conducted between 2021 and 2023 at two tertiary referral CI centers. For these studies, CI users were recruited through prior participation in in-person or remote studies, prior interest in participating in research, or word of mouth. Participants were 18 years or older, had one or two CIs with at least 6 months of CI experience, and were native American English speakers. All participants were postlingually deaf (defined as self-reported onset of hearing loss after age 12 years) and had no history of stroke, neurological disorder, or diagnosed cognitive impairment. Participants reported their age and occupational status at the time they completed the questionnaire. The study was approved under IRBs #2020H043 and #230093.

### 2.2. General Approach and Measures

Participants completed a lab-developed Speech Communication Questionnaire (SCQ) that was administered using the online platforms REDCap and Gorilla [41,42]. The SCQ was developed to assess several real-world speech and social communication experiences and challenges that are not currently captured by existing standardized and validated questionnaires. Specific questions and real-world challenges were developed based on clinical experience and prior research on CI outcomes, including aspects of communication environments and partners and downstream consequences of challenging listening conditions, such as experience of listening effort and fatigue, memory, and social interaction. The SCQ has not undergone formal psychometric validation (e.g., assessment of construct validity or reliability), and results derived from the use of this instrument should therefore be interpreted as exploratory. Since the SCQ focused on patient-reported communication priorities and real-world listening challenges rather than speech perception in standardized testing conditions, clinical speech recognition measures (e.g., CNC word scores) were not collected.

In addition to the SCQ, participants completed the Cochlear Implant Quality of Life (CIQOL) questionnaire, a validated patient-reported outcome measure designed to assess CI-related quality of life across multiple domains. For the present analyses, the Communication subdomain and Global score were examined to provide a standardized measure of self-reported communication ability and overall CI-related quality of life. CIQOL data were available for most participants and were used to contextualize SCQ findings.

For the current study, six questions relating to specific speech communication experiences or difficulties are reported: (1) Important communication partners; (2) Occupational status; (3) Specific conversational challenges; (4) Difficulty remembering unclear speech; (5) Specific tiring conversations; and (6) Avoiding difficult conversations. See Appendix A Table A1 for questions and coding.

### 2.3. Data Processing

Differences between the versions of the questionnaire used for the two separate studies were reconciled prior to data analysis. The number of participants providing a “yes” vs. “no” response was tallied to determine how many total specific challenges were selected from question 1 and how many total specific situations felt difficult or tiring from question 5. Question 4 asked participants whether they had trouble remembering what people said when they were difficult to understand. Responses were recoded into a binary “yes” or “no”; “yes” responses indicated some difficulty remembering unclear speech. All other responses were recoded to “No”. Question 6 asked participants, “Do you ever avoid talking to people because they are difficult to understand?” Responses were recoded into a binary “yes” or “no”, where “yes” included agreement or neutrality. All other responses were recoded as “No”. Dichotomization of the data was necessary for Questions 4 and 6 to ensure consistent comparisons across two versions of the SCQ questionnaire. Note that Appendix A Table A1 demonstrates the recoded versions of the questions.

### 2.4. Statistical Analysis

Chi-square and Fisher’s exact tests were implemented for statistical analysis of the binary variables (i.e., selection of “yes”/“no”) whereas the Mann–Whitney U test was used for non-binary variables (i.e., average number of specific conversational challenges and tiring conversations). Two sets of analyses were performed on the same dataset comparing age group—younger (<65 years) or older (≥65 years)—and occupational status (“working” or “retired”) based on self-reported age and occupational status. When performing statistical analysis on binary variables, Fisher’s exact tests were used if any of the frequencies within the comparison were below 5, and chi-square tests were used for frequencies of 5 or above. An alpha of 0.05 was set with *p* < 0.05 used for statistical significance. All statistical analyses were performed using RStudio (R version 4.5.1).

Age and occupational status were strongly associated (χ^2^ = 24.499, *p* < 0.001), with younger CI users primarily working and older CI users primarily retired (see Appendix A Table A2 for the distribution of age group and occupational status). Since this dependency produces substantial multicollinearity, and because subgroup sizes were uneven, multivariable regression models including both predictors would yield unstable estimates. Therefore, analyses were conducted in an exploratory and descriptive manner and were not intended to estimate causal relationships or independent effects.

Given the exploratory nature of this study and the modest sample size, formal corrections for multiple comparisons (e.g., Bonferroni or false discovery rate) were not applied, as these approaches can substantially increase Type II error and obscure potentially meaningful patterns in smaller samples. Additionally, since multiple statistical comparisons were conducted, the possibility of Type I error should be considered, particularly for results near the significance threshold. Findings should therefore be interpreted descriptively, with emphasis on effect size, directionality, and consistency across related outcomes rather than statistical significance and *p*-values alone.

### 2.5. Exploratory Analyses

To evaluate whether age and occupational status could be meaningfully examined jointly, we conducted exploratory descriptive analyses across four combined age–occupation groups (younger–working, younger–retired, older–working, older–retired). These analyses assessed the distribution of participants across age–occupation combinations in the SCQ data and the feasibility of joint modeling.

Exploratory checks revealed sparse representation in the younger–retired (5/35) and older–working (8/34) groups, as well as strong alignment between age and occupational status across the sample. Based on these patterns, we determined that multivariable modeling including both predictors would produce unstable coefficient estimates, inflated standard errors, and estimates heavily reliant on extrapolation from small cell counts.

Given these limitations, analyses treating age and occupational status jointly were not pursued further and are not reported in this manuscript. Accordingly, primary analyses examined age (younger vs. older) and occupational status (working vs. retired) separately, without combining these variables or estimating independent effects.

## 3. Results

### 3.1. Participant Demographics

Table 1 shows the demographic information for the 69 CI survey participants included in the analyses. The number of participants included for a specific characteristic was included under “N”, accounting for cases where a participant did not provide a response. Duration of deafness to CI was defined as the self-reported number of years from the onset of hearing loss to first CI surgery. Appendix A Table A3 and Table A4 show counts and percentages for all included survey data based on age group and occupational status, respectively.

### 3.2. Comparisons of Responses Based on Age Group and Occupational Status

**Figure 1:**
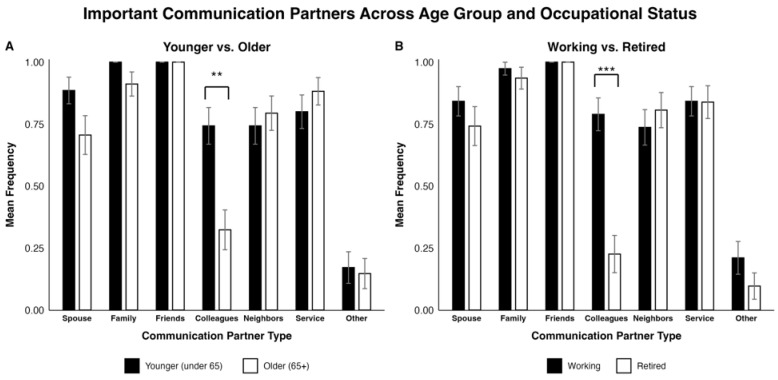
Side-by-side bar plots comparing participant responses on the importance of specific communication partners by (**A**) age group (younger vs. older) and (**B**) occupational status (working vs. retired). The values are reported as a mean frequency with standard error of the mean (SEM) as error bars. Significant differences are indicated as follows: ** = *p* < 0.01, *** = *p* < 0.001.

Regarding “Important communication partners”, younger CI users placed significantly greater importance on speaking with colleagues compared with older participants (χ^2^ = 10.566, *p* = 0.001; see Figure 1A). Similarly, working individuals emphasized greater importance of speaking with colleagues compared to retired individuals (χ^2^ = 19.604, *p* < 0.001; see Figure 1B). No significant differences were observed between age or occupational status groups for other communication partners (e.g., spouse, family, friends, neighbors, service, other).

**Figure 2:**
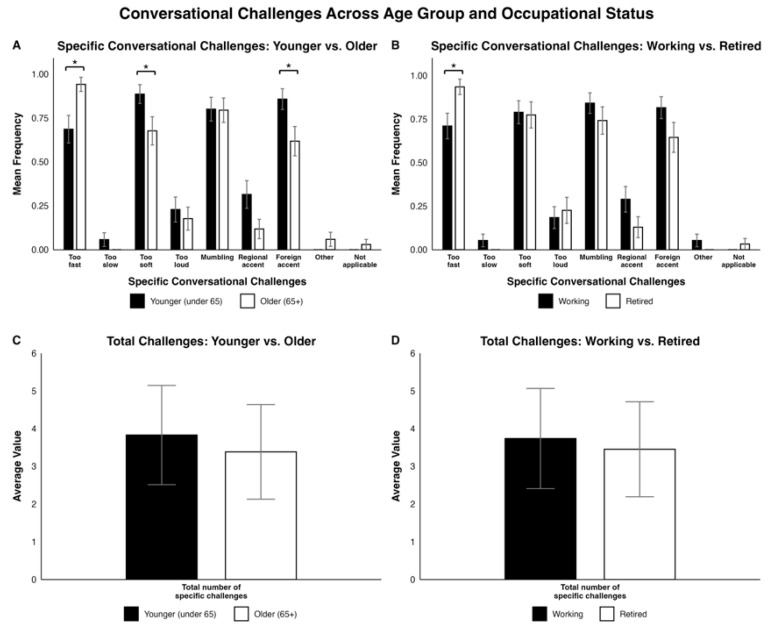
Side-by-side bar plots comparing participant responses on specific conversational challenges and average number of reported conversational challenges by (**A**,**C**) age group (younger vs. older) and (**B**,**D**) occupational status (working vs. retired). In (**A**,**B**), the values are reported as a mean frequency with SEM as error bars. In (**C**,**D**), the average value and standard deviation (SD) are reported. Significant differences are indicated as follows: * = *p* < 0.05.

Regarding “Specific conversational challenges”, younger participants reported fewer difficulties with fast speech (Fisher’s exact test, *p* = 0.012), but more difficulties with soft speech (Fisher’s exact test, *p* = 0.044) and understanding foreign accents (χ^2^ = 3.9635, *p* = 0.047) compared to older participants. Working participants reported fewer difficulties with fast speech (Fisher’s exact test, *p* = 0.028) compared to retired participants. All other comparisons between age groups and occupational statuses for specific conversational challenges were not statistically significant. Specific conversational challenges are displayed in Figure 2A,B. Overall, most survey respondents across all groups (>60%) had difficulties with conversations that involved fast speech, soft speech, mumbling, and foreign accents. Despite differences in specific conversational challenges, no statistically significant differences in the total number of specific challenges emerged by age group or occupational status (see Figure 2C,D).

**Figure 3:**
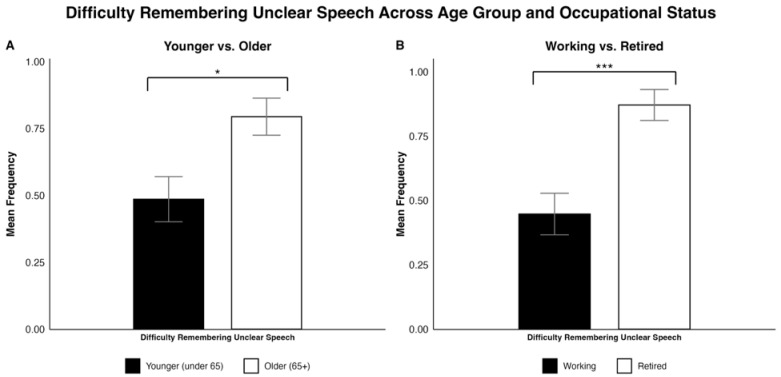
Side-by-side bar plots comparing participant responses on difficulty remembering unclear speech by (**A**) age group (younger vs. older) and (**B**) occupational status (working vs. retired). The values are reported as a mean frequency with SEM as error bars. Significant differences are indicated as follows: * = *p* < 0.05, *** = *p* < 0.001.

Regarding “Difficulty remembering unclear speech”, older CI users were more likely to have difficulty remembering someone’s words when they were difficult to understand (χ^2^ = 5.8276, *p* = 0.016; see Figure 3A). Similarly, retired adults were more likely to have difficulty remembering someone’s words when they were difficult to understand (Fisher’s exact test, *p* < 0.001; see Figure 3B).

**Figure 4:**
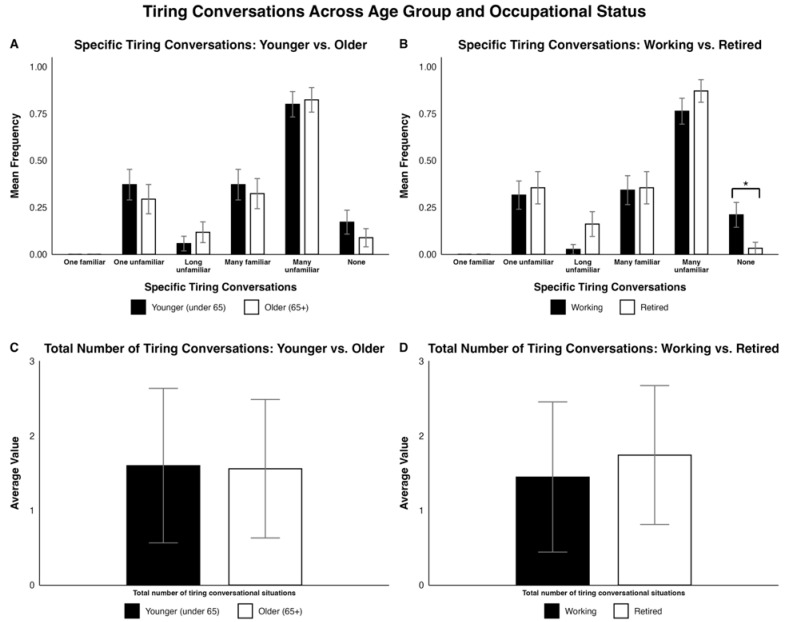
Side-by-side bar plots comparing participant responses on specific tiring conversations and average number of tiring conversations by (**A**,**C**) age group (younger vs. older) and (**B**,**D**) occupational status (working vs. retired). In (**A**,**B**), the values are reported as a mean frequency with SEM as error bars. In (**C**,**D**), the average value and SD are reported. Significant differences are indicated as follows: * = *p* < 0.05.

Regarding “Specific tiring conversations”, most participants (≥80%) in both younger and older groups reported becoming tired when talking to many unfamiliar individuals. However, no statistically significant differences were found between the young and old groups. Similarly, greater than 75% of working and retired participants reported becoming tired when talking to many unfamiliar individuals, though significantly more working participants indicated that none of the listed situations were tiring compared to retired participants (Fisher’s exact test, *p* = 0.035). None of the other specific tiring conversations that we tested revealed statistical differences between working and retired CI users. Across age groups and occupational statuses, a minority of participants (<40%) found the other conditions tiring. Furthermore, no participants found conversations with one familiar talker tiring. Finally, the total number of tiring conversational situations did not differ by age group or occupational statuses. See Figure 4A,C for age group and Figure 4B,D for occupational status.

**Figure 5:**
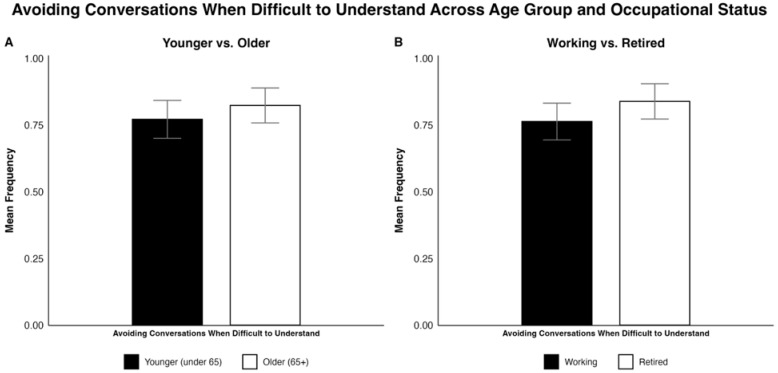
Side-by-side bar plots comparing participant responses on avoiding conversations perceived as difficult to understand by (**A**) age group (younger vs. older) and (**B**) occupational status (working vs. retired). The values are reported as a mean frequency with SEM as error bars.

Regarding “Avoiding difficult conversations”, there were no significant differences in avoidance of difficult conversations for age or occupation status. However, >75% of all survey participants reported avoiding conversations when they were hard to understand, as reported by Figure 5A,B.

### 3.3. CIQOL Outcomes

To assess whether group differences in reported communication experiences reflected differences in perceived communicative ability, Cochlear Implant Quality of Life (CIQOL) scores were compared across age group and occupational status using independent-samples *t*-tests. No statistically significant differences were observed between younger and older CI users for the CIQOL Communication subdomain score (Mean 46.9 vs. 47.5, respectively) or Global score (Mean 48.9 vs. 50.3, respectively), with all *p* > 0.05. Similarly, CIQOL Communication score (Mean 48.7 vs. 45.2, respectively) and Global score (Mean 50.9 vs. 47.8, respectively) did not differ significantly between working and retired participants, with all *p* > 0.05.

## 4. Discussion

This study defined the subjective communication experiences and challenges among adult CI users in everyday, real-world environments and explored how age and occupational status relate to these self-reported difficulties. Consistent with our hypotheses, results indicated both shared and distinct patterns in conversational difficulty, listening fatigue, and recall. Because age and occupational status may have been associated in this sample, all age- and occupation-related findings presented below should be interpreted as descriptive and overlapping rather than reflecting entirely independent effects.

### 4.1. Age-Related Experiences and Challenges

Younger individuals placed higher importance on speaking with colleagues compared to their respective counterparts. This trend might be partially explained by the average retirement age being around 64 for men and 62 for women [43], which is immediately below the cutoff of 65 years for the age group designation. Our dataset aligns with the literature—26 out of 34 individuals aged 65 and older were retired (76.5%) and only 5 out of 35 individuals younger than 65 were retired (14.3%). This idea is further supported by prior research on the general population (not specifically CI users) showing that retirees typically do not maintain communication with former coworkers [44]. These findings suggest that workplace-related auditory training may be particularly relevant for younger CI users, who are more likely to have an occupation.

Concerning specific speech challenges, younger individuals reported greater difficulty with soft speech, while older CI users struggled more with fast speech. Prior work has shown that normal-hearing older adults have more difficulties understanding fast speech, linked to age-related slowing of processing [45]. Additionally, Iwasaki et al. (2002) [26] showed that speech rate negatively impacted CI users but did not directly compare age groups. These findings align with our data, suggesting that training CI users, specifically older CI users, to process fast speech may be a valuable rehabilitative target. Younger CI users may have relatively more difficulties with soft speech because conversational partners might assume that they do not have trouble hearing, so they are less aware of their soft speech. In contrast, with older CI users, people might assume they are hard of hearing by default and talk louder (e.g., “elderspeak”) [46]. Clinicians may counsel younger CI users to be more vocal about their hearing loss to minimize difficulties with soft speech.

Interestingly, we found that younger CI users reported greater difficulty than older CI users in understanding foreign-accented speech. Previous research in non-CI adults had typically found no difference across ages [47] or increased difficulties [48] for older adults. We speculate that younger CI users may spend more time talking to people with foreign accents (e.g., workplace, traveling, etc.) than older CI users, which could influence their responses. These findings may also indicate that foreign-accented speech perception could be a priority target in auditory training for younger CI users.

Despite these differences, the total number of specific challenges faced by younger and older participants was similar, suggesting that all CI users face multiple communication barriers even with long-term CI use. Several environments—such as speech that is too fast, too soft, spoken with a foreign accent, and mumbled speech—were frequently cited as challenging by both age groups. Thus, there may be merit in addressing these difficulties for all adult CI users.

Our analyses revealed that younger CI users reported fewer difficulties with remembering unclear speech compared to older CI users. A multitude of factors could contribute to this finding, such as the effects of age on neurocognitive top-down processing or memory [49,50,51]. It may therefore be critical for older CI users to ask their conversational partners to repeat themselves more frequently or convey their thoughts in an alternative manner.

Greater than 75% of both younger and older participants reported experiencing tiredness when speaking with unfamiliar individuals and a tendency to avoid conversations when they were difficult to understand. These behaviors align with current listening effort models, which posit that sustained cognitive resources are required for speech perception in challenging environments [19,25,52,53]. High listening effort can lead to the avoidance of difficult conversations and fatigue, potentially reducing social engagement and contributing to feelings of isolation. Recognizing these experiences is important for clinical counseling and rehabilitation, as targeted interventions—such as group communication exercises, strategies to request clarification, and paced speech training—may help CI users maintain social participation and reduce fatigue during everyday interactions. By understanding why these situations are tiring and difficult, medical professionals can empower and train CI users via targeted clinical exercises to improve their outcomes in similar situations.

To assess whether age-related differences in reported communication experiences might reflect differences in perceived communicative ability, we examined scores from the Cochlear Implant Quality of Life (CIQOL) questionnaire. No significant age-related differences were observed for the CIQOL Communication subdomain or Global scores, indicating comparable self-reported communication ability across younger and older CI users. Thus, although clinical speech recognition measures (e.g., CNC word scores) were not available for the current sample, the observed age-related differences in specific communication challenges and priorities are unlikely to be driven solely by differences in communicative ability alone.

### 4.2. Occupational Status-Related Experiences and Challenges

The differences in occupational status groups (working vs. retired) followed similar trends to the age group comparisons, likely due to their overlapping influence. Notably, CIQOL Communication and Global scores also did not differ significantly between working and retired participants, suggesting that these occupational differences in communication priorities and challenges are unlikely to be driven by communicative ability alone.

Working individuals placed much greater importance on speaking with colleagues compared to retired individuals, reflecting workplace communication demands. Additionally, like older CI users, retired CI users had more difficulty with fast speech compared to working users. The specific association between retired individuals and understanding fast speech has not been explored well in the literature, both for CI and non-CI users. However, Shvartzman et al. (2022) [54] acknowledges that sensory and cognitive factors, as well as rapid perceptual learning—the ability to rapidly adapt to changes in the auditory environment—can influence speech understanding in older adults. In addition, Xue et al. (2018) [55] demonstrated that retirement may increase the risk of cognitive decline, which negatively affects verbal memory function. Thus, it could be argued that exposure to diverse conversations in the workplace supports rapid perceptual learning, leading to better perception of fast speech in working adults compared to retired adults. To maintain cognitive flexibility, clinicians could encourage retired CI users to engage in diverse conversations and cognitively stimulating activities.

Broadly, the total number of specific conversational challenges did not differ between working and retired individuals. Similar to the age group comparison, this finding might suggest that both working and retired CI users could benefit from addressing the major difficulties in conversations (i.e., too fast, too soft, mumbling, and foreign accent), regardless of occupational status.

On another note, working CI users reported fewer difficulties with remembering unclear speech compared to retired participants. This finding may align with Xue et al. (2018) [55], who stated that retirement generally decreases verbal memory function through cognitive decline. Interestingly, it also parallels our data indicating that younger CI participants had fewer difficulties with remembering unclear speech compared to older CI participants, suggesting that it is possible that there is an association between the two findings.

We also found that a significantly larger proportion of working CI users reported not experiencing tiring conversations compared to retired CI users. This suggests that retired CI users may be more prone to fatigue during conversations, which naturally increases with age and social isolation, making it difficult to address clinically [25,56]. Additionally, >75% of working and retired participants became tired when talking to many unfamiliar individuals while a minority of participants (<40%) found the other conditions tiring. Thus, it is especially important to train CI users to hear others in environments involving many unfamiliar individuals, regardless of occupational status. Furthermore, similar to the age group comparison, >75% of working and retired participants avoided conversations when they were difficult to understand. Addressing this challenge is difficult because communication breakdowns can feel emotionally taxing or embarrassing to CI users; nevertheless, encouraging them to ask their conversational partners to repeat themselves may improve their social interactions [21,57,58]. As suggested with the age-related analysis of communication experiences and challenges, similar interventions—group communication exercises, strategies to request clarification, and paced speech training—might be considered to address these issues of unclear speech and listening fatigue in accordance with existing listening effort models.

### 4.3. Overlap Between Age and Occupational Status

Given the strong association between age and occupational status in this sample, it is impossible to isolate their individual contributions to self-reported communication experiences and challenges for postlingually deafened adult CI users. Because most individuals retire around 62–64 years [43], our dataset included few older working adults or younger retirees, limiting our ability to disentangle these effects.

There are at least two possible mechanisms that may contribute our findings: (1) changes in social interactions attributed to occupational status and (2) age-related cognitive decline. Loss of social interactions in the workplace may reduce exposure to diverse speech environments, which are critical for maintaining speech communication skills [55,59]. Although retired individuals do not necessarily have a smaller social circle than working individuals, transitioning from working to retired leads to loss of a consistent speech environment that could normally be conducive to resilience against subjective communication challenges. Additionally, seeing a significantly low number of retired CI participants choosing “none” when asked about tiring situations could indicate that retired individuals engage with fewer individuals in general (and thus do not feel tired). This reduced interaction could contribute to the difficulties with fast speech and memory observed in older and retired individuals since their language processing capabilities might decline.

Age-related cognitive changes, including declines in perceptual learning, top-down processing, and listening fatigue are well-documented [25,49,50,51,54,55,56]. Older adults also tend to have similar or worse objective hearing scores compared to younger adults [60,61]. These factors may compound communication difficulties, particularly for CI users [25,49,50,51,54,55,56]. Clinicians could be encouraged to identify and address these challenges early to obtain the best possible outcomes for CI users [62,63]. Additionally, maintaining a more robust and consistent communication environment, which is more common among younger and working individuals, may help mitigate these effects [59,64].

Because these mechanisms were confounded in our sample, we could not determine whether observed differences reflected age, occupational status, or their interaction. Future research targeting balanced representation across these categories is necessary to clarify their independent contributions. Regardless of which factor has a greater impact, meeting patients where they are and addressing their needs on an individualized basis is critical. This study can serve as a tool to know the types of questions to ask CI users and the trends to expect based on age and occupational status; however, the path toward addressing their clinical and personal goals is unique to each patient.

### 4.4. Clinical Implications

The findings of this study may offer guidance for clinical counseling and rehabilitation for adults who are pre- or post-implantation. Pre-CI counseling may benefit from discussing potential communication challenges based on age and occupational status. Based on our findings, younger and working CI users may encounter difficulties with soft speech and foreign-accented speech while also valuing conversations with colleagues. Older and retired CI users may face challenges with fast speech and difficulty remembering unclear speech. All adult CI users might need to be warned about potential conversation-related fatigue with many unfamiliar individuals. These discussions may help patients develop realistic expectations for everyday listening and facilitate early conversations with the clinical team about strategies to overcome these challenges.

Post-CI counseling and rehabilitation can target these areas through auditory training tailored to individual challenges, strategies for managing listening effort, and exercises to support social participation. Younger and working CI users may require additional focus on workplace-related communication and simulations for comprehending soft and foreign-accented speech. Older and retired users may benefit from exercises that train for fast speech comprehension and remembering unclear speech. Across all adult CI users, interventions such as paced speech exercises, group communication activities, and strategies for requesting speech clarification may improve functional outcomes by addressing listening fatigue and conversations that are difficult to understand. While this study did not directly evaluate counseling practices, the findings highlight opportunities for clinicians to anticipate potential challenges and personalize support for CI users throughout their rehabilitation.

### 4.5. Limitations and Future Directions

One limitation in this study is the relatively small sample size, which may impact the strength and generalizability of the statistical findings. However, the detailed nature of the questionnaires allowed us to examine the specific communication challenges of the sample population more closely, and a sample size of 69 participants is consistent with or even larger than most similar studies of adult CI users. Second, most surveys were completed around the peak of the COVID-19 pandemic, when there was a prevalence of masks. Given that many participants relied on lip reading for speech comprehension, the data may not be entirely representative of current CI users. However, it should be noted that most of the questions included in the survey examined more general abilities and challenges and did not specifically assess the impact of wearing masks or COVID-related restrictions. Third, this study did not incorporate a control group, but most studies investigating factors contributing to individual differences within one group of participants do not include control groups, as the goal is to determine what contributes to variability in abilities, outcomes, or challenges within that group. Fourth, the SCQ has not undergone formal psychometric validation, including assessment of construct validity or reliability. Thus, while the SCQ enabled the investigation of real-world communication experiences not captured by existing standardized measures, results derived from this instrument should be interpreted as exploratory and hypothesis-generating rather than confirmatory. Future work should include formal validation of the SCQ to establish its measurement properties. Finally, we acknowledge that other communication challenges exist beyond our survey, such as speech-in-noise perception. Despite these limitations, the data presented align with prior studies and may provide valuable information about the communicative experiences and challenges across age groups and occupational statuses that can be further explored.

Many studies have focused on quantitative clinical speech outcomes, and an increasing number are now analyzing quality of life and subjective experiences. However, few studies have evaluated the combination of quantitative speech perception outcomes with subjective experiences to understand real-world communication challenges. Future studies could explicitly integrate clinical speech recognition measures (e.g., CNC word scores) with patient-reported communication priorities to clarify how objective performance relates to perceived challenges in everyday listening environments. Additionally, it would be beneficial to disentangle the impacts that age and occupational status have on the experiences and challenges of CI users, given that they are closely intertwined in this study. Further exploration would allow clinicians to better utilize demographic information to tailor exercises and treatments for CI users.

## 5. Conclusions

These findings highlight nuanced, real-world experiences and challenges that CI users face and suggest that age and occupational status may shape specific aspects of speech perception beyond what standardized tests typically capture. It is possible that one plays a greater role than the other in the challenges and experiences of CI users, but both are influential to an individual’s outcomes. Ultimately, these insights may be helpful in informing individualized counseling, rehabilitation strategies, and expectations in clinical practice.

## Figures and Tables

**Table 1:** Demographic Information for Included CI Survey Participants.

	N	Value
Age	69	
≥65 years old (Older)		34
<65 years old (Younger)		35
Mean ± SD		61.6 ± 13.3 years
Occupational Status	69	
Working		38
Retired		31
Sex	68	
Male		23
Female		45
CI Configuration	67	
Unilateral		11
Bilateral		29
Bimodal (unilateral CI and contralateral HA)		27
Age at First CI (years)	66	
Mean ± SD		51.8 ± 15.8 years
Duration of Deafness to CI	67	
Mean ± SD		17.3 ± 17.7 years

SD indicates standard deviation; CI, cochlear implant; HA, hearing aid.

## Data Availability

The raw data supporting the conclusions of this article may be made available by the authors on request.

## Abbreviations

The following abbreviations are used in this manuscript:

CI	Cochlear implant
CIQOL	Cochlear Implant Quality of Life
WHO	World Health Organization
SCQ	Speech Communication Questionnaire
SD	Standard deviation
SEM	Standard error of the mean

## Appendix A

**Table A1:** Questions and Answer Choices in Speech Communication Questionnaire.

Question	Answer(s)
Q1. With which groups of people is it important for you to communicate using spoken conversations? You can select more than one.	Spouse/partner Other family Friends Colleagues Neighbors Service people (e.g., at the grocery store) Other
Q2. Are you retired? What is (or was) your occupation?	Yes No Open-Ended Response for Occupation
Q3. In general, which of the following features of speech make communication difficult for you?	Too fast Too slow Too soft Too loud Pronunciation not clear (e.g., mumbling) Regional accent Foreign accent Other ______ None of the above
Q4. Do you have trouble remembering what people say, especially when they are difficult to understand?	Yes No
Q5. Which situations are difficult or tiring for you? You may select more than one response.	Having a conversation with one familiar talker (e.g., family, friends) Having a conversation with one unfamiliar talker (e.g., service person) Having a conversation with one unfamiliar talker (e.g., service person) after you have talked with him/her for a while (e.g., a long conversation) Having a conversation with more than one familiar talker Having a conversation with more than one unfamiliar talker None of the above
Q6. Do you ever avoid talking to people because they are difficult to understand?	Yes No

**Table A2:** Distribution of Participants Across Age Group and Occupational Status.

	Occupational Status	
**Age Group**	Working	Retired
Younger (<65)	30	5
Older (≥65)	8	26

**Table A3:** Counts and Percentages of Values for Younger vs. Older Participants.

Age	Younger (<65) N = 35		Older (≥65) N = 34	
	Yes (%)	No (%)	Yes (%)	No (%)
**Important Communication Partners**				
Spouse	31 (88.6)	4 (11.4)	24 (70.6)	10 (29.4)
Family	35 (100)	0 (0)	31 (91.2)	3 (8.8)
Friends	35 (100)	0 (0)	34 (100)	0 (0)
Colleagues	26 (74.3)	9 (25.7)	11 (32.4)	23 (67.6)
Neighbors	26 (74.3)	9 (25.7)	27 (79.4)	7 (20.6)
Service	28 (80.0)	7 (20.0)	30 (88.2)	4 (11.8)
Other	6 (17.1)	29 (82.9)	5 (14.7)	29 (85.3)
**Specific Challenges**				
Too fast	24 (68.6)	11 (31.4)	32 (94.1)	2 (5.9)
Too slow	2 (5.7)	33 (94.3)	0 (0)	34 (100)
Too soft	31 (88.6)	4 (11.4)	23 (67.6)	11 (32.4)
Too loud	8 (22.9)	27 (77.1)	6 (17.6)	28 (82.4)
Mumbling	28 (80.0)	7 (20.0)	27 (79.4)	7 (20.6)
Regional accent	11 (31.4)	24 (68.6)	4 (11.8)	30 (88.2)
Foreign accent	30 (85.7)	5 (14.3)	21 (61.8)	13 (38.2)
Other	0 (0)	35 (100)	2 (5.9)	32 (94.1)
Not Applicable	0 (0)	35 (100)	1 (2.9)	33 (97.1)
**Tiring Conversations**				
One familiar talker	0 (0)	35 (100)	0 (0)	34 (100)
One unfamiliar talker	13 (37.1)	22 (62.9)	10 (29.4)	24 (70.6)
Long conversation with unfamiliar talker	2 (5.7)	33 (94.3)	4 (11.8)	30 (88.2)
Many familiar talkers	13 (37.1)	22 (62.9)	11 (32.4)	23 (67.6)
Many unfamiliar talkers	28 (80.0)	7 (20.0)	28 (82.4)	6 (17.6)
None	6 (17.1)	29 (82.9)	3 (8.8)	31 (91.2)
**Avoiding Conversations When They Are Difficult to Understand**	27 (77.1)	8 (22.9)	28 (82.4)	6 (17.6)
**Trouble Remembering Unclear Speech**	17 (48.6)	18 (51.4)	27 (79.4)	7 (20.6)
	**Average Value**			
**Total Number of Specific Challenges in Conversations**	3.83		3.38	
**Total Number of Tiring Conversational Situations**	1.60		1.56	

**Table A4:** Counts and Percentages of Values for Working vs. Retired Participants.

Occupational Status	Working N = 38		Retired N = 31	
	Yes (%)	No (%)	Yes (%)	No (%)
**Important Communication Partners**				
Spouse	32 (84.2)	6 (15.8)	23 (74.2)	8 (25.8)
Family	37 (97.4)	1 (2.6)	29 (93.5)	2 (6.5)
Friends	38 (100)	0 (0)	31 (100)	0 (0)
Colleagues	30 (78.9)	8 (21.1)	7 (22.6)	24 (77.4)
Neighbors	28 (73.7)	10 (26.3)	25 (80.6)	6 (19.4)
Service	32 (84.2)	6 (15.8)	26 (83.9)	5 (16.1)
Other	8 (21.1)	30 (78.9)	3 (9.7)	28 (90.3)
**Specific Challenges**				
Too fast	27 (71.1)	11 (28.9)	29 (93.5)	2 (6.5)
Too slow	2 (5.3)	36 (94.7)	0 (0)	31 (100)
Too soft	30 (78.9)	8 (21.1)	24 (77.4)	7 (22.6)
Too loud	7 (18.4)	31 (81.6)	7 (22.6)	24 (77.4)
Mumbling	32 (84.2)	6 (15.8)	23 (74.2)	8 (25.8)
Regional accent	11 (28.9)	27 (71.1)	4 (12.9)	27 (87.1)
Foreign accent	31 (81.6)	7 (18.4)	20 (64.5)	11 (35.5)
Other	2 (5.3)	36 (94.7)	0 (0)	31 (100)
Not Applicable	0 (0)	38 (100)	1 (3.2)	30 (96.8)
**Tiring Conversations**				
One familiar talker	0 (0)	38 (100)	0 (0)	31 (100)
One unfamiliar talker	12 (31.6)	26 (68.4)	11 (35.5)	20 (64.5)
Long conversation with unfamiliar talker	1 (2.6)	37 (97.4)	5 (16.1)	26 (83.9)
Many familiar talkers	13 (34.2)	25 (65.8)	11 (35.5)	20 (64.5)
Many unfamiliar talkers	29 (76.3)	9 (23.7)	27 (87.1)	4 (12.9)
None	8 (21.1)	30 (78.9)	1 (3.2)	30 (96.8)
**Avoiding Conversations When They Are Difficult to Understand**	29 (76.3)	9 (23.7)	26 (83.9)	5 (16.1)
**Trouble Remembering Unclear Speech**	17 (44.7)	21 (55.3)	27 (87.1)	4 (12.9)
	**Average Value**			
**Total Number of Specific Challenges in Conversations**	3.74		3.45	
